# Increased Risk of Q151M and K65R Mutations in Patients Failing Stavudine-Containing First-Line Antiretroviral Therapy in Cambodia

**DOI:** 10.1371/journal.pone.0073744

**Published:** 2013-08-28

**Authors:** Janin Nouhin, Yoann Madec, Nicole Ngo-Giang-Huong, Laurent Ferradini, Eric Nerrienet

**Affiliations:** 1 HIV/Hepatitis Unit, Institut Pasteur in Cambodia, Phnom Penh, Cambodia; 2 Université Paris Diderot, Sorbonne Paris Cité, Paris, France; 3 Emerging Disease Epidemiology Unit, Department of Infection and Epidemiology, Institut Pasteur, Paris, France; 4 Institut de Recherche pour le Développement (IRD UMI 174), Paris, France; 5 Faculty of Associated Medical Sciences, Chiang Mai University, Chiang Mai, Thailand; 6 Harvard School of Public Health, Boston, Massachusetts, United States of America; 7 FHI 360, Phnom Penh, Cambodia; 8 Regulation of Retroviral Infection Unit, Department of Virology, Institut Pasteur, Paris, France; University of Pittsburgh, United States of America

## Abstract

**Background:**

Multi-nucleos(t)ide resistance (MNR) mutations including Q151M, K65R mutations, and insertion at codon 69 of HIV-1 reverse transcriptase coding region may confer resistance to all molecules of nucleos(t)ide reverse transcriptase inhibitors (NRTI). The presence of these mutations is an emerging problem compromising non-nucleoside reverse transcriptase inhibitors and protease inhibitors-based therapies. Furthermore, factors associated with selection of these mutations are still not well defined. The current study aimed to evaluate the frequency and to characterize factors associated with the occurrence of multi-nucleos(t)ide resistance mutations among HIV-1 infected patients failing recommended first-line antiretroviral regimens in Cambodia.

**Methodology/Principal Finding:**

This is a retrospective analysis of HIV-1 drug resistance genotyping of 520 HIV-1 infected patients in virological failure (viral load > 250 copies/mL) while on first-line antiretroviral therapy in Cambodia with at least one reverse transcriptase inhibitor resistance associated mutation. Among these 520 patients, a total of 66 subjects (66/520, 12.7%) presented ≥1 MNR mutation, including Q151M, K65R, and Insert69 for 59 (11.3%), 29 (5.6%), and 2 (0.4%) patients, respectively. In multivariate analysis, both Q151M (p = 0.039) and K65R (p = 0.029) mutations were independently associated with current stavudine- compared to zidovudine-use.

**Conclusion:**

Such selection of mutations by stavudine drastically limits the choice of antiretroviral molecules available for second-line therapy in resource-limited settings. This finding supports the World Health Organization’s recommendation for stavudine phase-out.

## Introduction

Q151M, K65R substitutions, and insertions at codon 69 (Insert69) in the reverse transcriptase encoding region of HIV-1 genome confer resistance to a large range of nucleos(t)ide reverse transcriptase inhibitors (NRTIs) and are thus called multi-nucleos(t)ide resistance (MNR) mutations. Q151M confers resistance to all NRTIs, except tenofovir (TDF) [1]. K65R confers resistance to stavudine (d4T) and TDF and possibly to lamivudine/emtricitabine (3TC/FTC), didanosine (ddI) and abacavir (ABC) [2], [3], [4], [5], while Insert69 confers resistance to all NRTIs available today [6], [7], [8]. These mutations dramatically compromise subsequent antiretroviral (ARV) regimens.

Factors associated with selection of these mutations are still not well defined. d4T/ddI dual therapy has been associated with the occurrence of Q151M and K65R [9], [10], [11], as well as young age, low CD4^+^ T-cell count, high HIV RNA viral load (VL) and experience of more than two ARV regimens before resistance genotyping testing [12], [13]. However, these studies mostly enrolled patients under dual therapies. Few data on Highly Active Antiretroviral Therapy (HAART) failure in resources limited setting are available [5], [13]. Our study aimed to evaluate the frequency and to identify factors associated with the occurrence of MNR mutations among HIV-1 CRF01_AE infected patients failing recommended first-line HAART in Cambodia.

## Methods

### Ethics statement

The current study was a retrospective and anonymous analysis of data collected in the context of routine care of HIV infected patients. Data was stored in a database of HIV/Hepatitis Laboratory, Institut Pasteur in Cambodia situated in Phnom Penh (the capital city of Cambodia). This kind of analysis complies with the Cambodian ethical guidelines for exemption from ethical approval requirement [14].

### Study design

We conducted a retrospective and observational study using data routinely collected between December 2004 and January 2011 from 9018 HIV-1 infected patients, on first-line ARV regimen, and in the context of routine care. All samples of these 9018 individuals were referred from many parts of Cambodia (including the capital city and 7 provinces).

In agreement with the World Health Organization (WHO) recommendation, the Cambodian national guidelines have recommended d4T, 3TC, and nevirapine (NVP) as the standard first-line ARV regimen and zidovudine (AZT), TDF, ABC, efavirenz (EFV), and/or protease inhibitors in case of drug side-effect or interaction. Laboratory monitoring included CD4^+^ T-cell count every six months whereas HIV-1 RNA VL and drug resistance genotyping were carried out to confirm clinical and/or immunological failure [15], [16].

All specimens were assessed for HIV-1 RNA VL testing and drug resistance genotyping in HIV/Hepatitis Laboratory of Institute Pasteur in Cambodia which was (at the time of the study) centralizing all virological tests for HAART monitoring.

HIV-1 RNA VL was performed on stored (−80°C) plasma specimens using the G2 Generic HIV-1 VL ANRS kit (Biocentric, Bandol, France) [17]. If the HIV-1 RNA VL is above the threshold (VL > 250 copies/mL), the reverse transcriptase inhibitor (RTI) resistance genotyping test was performed using bulk sequencing of HIV-1 RT coding region according to complete sequencing procedures and primer sequences described by ANRS (Agence Nationale de Recherche sur le SIDA et les hepatites virale – French national Agency for research on AIDS and viral hepatitis) working group [18].

## Results and Discussion

Of 9018 patients referred to our laboratory, 8304 (92.1%) had undetectable HIV-1 RNA VL. This rate represented a good virological outcome and was consistent with studies previously conducted in Cambodia amongst HIV-1 infected patients after one to three years of first-line ARV regimen [19], [20], [21], [22]. Seven hundred and fourteen patients had detectable HIV-1 RNA VL. Among these, 194 patients presented no resistance associated mutation (RAM) and were excluded from the present analysis as we assumed they were not adherent to the ARV treatment [23]. Finally, 520 subjects infected with HIV-1 CRF01_AE strains and presenting at least one RTI RAM were included in this analysis.

Their median age was 28 years [Inter Quartile Range (IQR): 9-38] at the time of sampling, and 233 (44.8%) patients were children (≤15 years) followed-up at the National Pediatric Hospital (Table 1). Median duration on current first-line regimen was 30 months [IQR: 18-44], and median HIV-RNA VL was 4.5log_10_ copies/mL [IQR: 4.0-5.1]. Three hundred and twenty eight patients (63%) (212 children and 116 adults) were receiving d4T-containing regimens and the remaining was under an AZT-containing regimen.

**Table 1:** Multivariate analysis of factors associated with the occurrence of Q151M and K65R mutations.

		Q151M			K65R		
	All subjects (n = 520)	Patients (n = 59)	Adjusted OR† [95% CI]‡	*p value*	Patients (n = 29)	Adjusted OR† [95% CI] ‡	*p value*
**Male gender**, n (%)	330 (63.5)	42 (71.2)			17 (58.6)		
**Age group**, n (%)				0.32			
Children (≤ 15 years)	233 (44.8)	33 (55.9)	1		17 (58.6)		
Adult (> 15 years)	287 (55.2)	26 (44.1)	0.73 [0.39 – 1.36]		12 (41.4)		
**HIV RNA VL level,** n (%)				<0.001			0.002
≤ 4.5log_10_ copies/mL	264 (50.8)	15 (25.4)	1		7 (24.1)	1	
> 4.5log_10_ copies/mL	256 (49.3)	44 (74.6)	4.02 [2.13 – 7.59]		22 (75.9)	3.97 [1.64 – 9.61]	
**Current 1^st^ line regimen**, n (%)				0.039			0.029
D4T containing	328 (63.1)	47 (79.7)	2.21 [1.05 – 4.65]		24 (82.8)	3.02 [1.12 – 8.13]	
AZT containing	192 (36.9)	12 (20.3)	1		5 (17.2)	1	
**Duration under current 1^st^ line,** n (%)				0.012			0.020
≤ 30 months	269 (51.7)	24 (40.7)	1		19 (65.6)	1	
> 30 months	251 (48.3)	35 (59.3)	2.11 [1.18 – 3.76]		10 (34.4)	2.60 [1.16 – 5.83]	

† Odd ratio estimated using logistic analysis.

‡ 95% Confident interval.

According to the ANRS algorithm (version 19) [18], 498 (95.8%) patients were resistant to at least 3 RTIs and 339 (65.2%) to 4 RTIs. The most frequently detected mutations were: M184I/V (92.3%), Y181C/I/V (47.1%), T215I/C/F/N/S (38.8%), D67E/G/N (37.3%), K103N/R/S (33.9%), and G190A/Q/S (32.5%). Regarding MNR mutations, a total of 66 subjects (66/520, 12.7%) presented ≥1 MNR mutation, including Q151M, K65R, and Insert69 for 59 (11.3%), 29 (5.6%), and 2 (0.4%) patients, respectively.

To identify factors associated with the presence of Q151M and K65R mutations, univariate and multivariate logistic regression analysis were performed using the following variables: gender, age group (≤ or >15 years), HIV-1 RNA VL level (≤ or >4.5log_10_ copies/mL), current first-line regimen (d4T- or AZT-containing) and duration (≤ or >30 months) at sampling time.

In univariate analysis, Q151M was significantly more frequent in patients on d4T-containing regimens (14.3%) than in those on AZT-containing regimens (6.3%) (p = 0.005). A high HIV-1 RNA level was also significantly associated with Q151M (p<0.0001). Q151M mutation tended to be associated with age ≤15 years (p = 0.069) and duration >30 months under current regimen (p = 0.071). In multivariate analysis, independent risk factors for Q151M were d4T-containing regimen (Odds ratio (OR) [95% confidence interval (CI)]: 2.21 [1.05−4.65]), high HIV-1 RNA level (OR [95% CI]: 4.02 [2.13−7.59]), and duration >30 months under current first-line regimen (OR [95% CI]: 2.11 [1.18−3.76]) (table 1).

The occurrence of K65R was significantly associated with d4T-containing regimens (p = 0.017) and high HIV-1 RNA level (p = 0.003), while there was a trend with duration >30 months under current first-line regimen (p = 0.054). In multivariate analysis, independent risk factors for K65R were d4T-containing regimen (OR [95% CI]: 3.02 [1.12−8.13]), high HIV-RNA level (OR [95% CI]: 3.97 [1.64−9.61]) and duration >30 months under current first-line regimen (OR [95% CI]: 2.6 [1.16−5.83]) (table 1).

In multivariate analysis, besides higher HIV-1 RNA VL level, probably associated with longer duration of failure, we found that d4T-use remained independently associated with the presence of MNR mutations (either Q151M or K65R). Finally, the Q151M and K65R mutations were positively associated to each other. Indeed, K65R was detected in 12/59 (20.3%) patients harboring Q151M compared to 3.9% of patients without Q151M (χ_2_ test, p<0.001). This result is consistent with previous study conducted in Italy [24]. In our study, other NRTI-RAMs known to be associated with Q151M-mediated multi-nucleoside resistance including A62V, V75I, F77L, and F116Y [12], were also significantly more common in patients harboring the Q151M mutation (p<0.001).

Other studies have reported a link between the use of d4T/ddI combination and the selection of MNR mutations, in particular among patients infected by HIV-1 non B genotype [9], [10], [11], [13], [25]. However, in our study, no patients received ddI at the time of genotyping. Indeed, children could not be prescribed ddI as first-line regimen under Cambodian protocol. Thus, our data strongly suggests that d4T *per se* is directly associated with the occurrence of both Q151M and K65R mutations.

Our retrospective study has some limitations. First, history of ARV treatment was incomplete for all patients included in the study. The initial first-line regimen recommended in Cambodia was based on d4T and it is very likely that a significant proportion of adults under AZT at the time of genotyping had initiated a prior d4T-based first-line regimen which was then changed to AZT due to the occurrence of adverse events. This might explain the presence of Q151M mutation among some patients under AZT regimen (6.3%) at the time of genotyping. As reported recently (2012) by Phan et al., in a follow-up conducted between 2003 and 2010 in the Sihanouk-Hospital-Center-of-Hope in Phnom Penh, Cambodia, d4T was discontinued and switched to AZT in around 48% of patients due to d4T-toxicity [26] while no AZT to d4T switch was reported. Due to similar setting, it is very likely that the proportion of d4T to AZT (and AZT to d4T) switches in our study population are very close to those found by Phan et al. As consequence, we may have underestimated the risk of MNR mutations due to d4T-use since some have been erroneously attributed to AZT-use. Second, we were not able to document duration of treatment failure, and adherence levels to ARV drugs. It is thus possible that other factors not analyzed herein were not taken into account with the occurrence of MNR mutations.

## Conclusion

In conclusion, our data suggests that the administration of d4T *per se* is associated with the emergence of both Q151M and K65R MNR mutations which confer resistance to a large range of NRTIs. It is important to note that the effect of d4T is observed even after adjusting for HIV-1 RNA VL. Selection of such mutations is a critical issue in resources-limited settings where NRTI molecules available for second-line regimen are still limited. Although the primary reasons for the 2010 WHO’s recommendation to reduce or abandon the use of d4T as part of first-line ARV regimen in resource-limited settings were related to excessive toxicity [27], our findings further support this guidance due to association with mutations which render the virus resistant to most or all of NRTI. However, the transition to alternative regimens still appears difficult and d4T remains widely used in such settings because of its affordability as a low-cost generic fixed-dose combinations. Recently, the Cambodian HIV National Program committed to move out d4T from its recommended first-line ARV regimen in the national guideline.

### Nucleotide accession number

Five hundred and twenty nucleotide sequence of CRF01_AE HIV-1 reverse transcriptase coding region included in the current analysis have been submitted to Genkbank under the accession numbers KF292310 to KF292829.
